# In this month’s Bulletin

**DOI:** 10.2471/BLT.22.001222

**Published:** 2022-12-01

In the editorial section, Nathalie Roebbel et al. (750) outline global research priorities for urban health. Hans Henri P Kluge et al. (751) introduce digital transformation programmes in the WHO Region of Europe. 

Gary Humphreys (754–755) reports on an initiative to replace tobacco cultivation with healthier crops in Kenya. In the interview section, Maria Munir Yusuf talks to Humphreys (756–757) about her work in Ethiopia to provide shelter, care and livelihoods for women who survive gender-based violence.

## Bangladesh, Brazil, Cambodia, China, Ethiopia, Greece, India, Italy, Kenya, Madagascar, Nigeria, Pakistan, Rwanda, Senegal, South Africa, Thailand, Uganda, Viet Nam

### Multidrug-resistant infections in neonates

Phoebe CM Williams et al. (798–807) propose an antibiotic development list for priority pathogens.

## Brazil, India, Peru, Portugal, South Africa, United States of America

### Managing ototoxicity

Michael M Lindeborg et al. (789–797) argue for standard definitions and better treatment options.

## Burkina Faso

### Preventing mother-to-child transmission of HIV 

Béninwendé Leticia Delphine Sakana et al. (769–776) evaluate use of a routine immunization visit.

## China

### Antineoplastics

Mengyuan Pan et al. (758–768) assess national procurement data.

## Ethiopia, Haiti, India, Kenya, Myanmar, Nepal, Nigeria, Peru, Thailand, Zambia

### Treating tuberculosis in children and adolescents

Courtney M Yuen et al. (777–788) review the evidence for integrated care models.

## Global

### Improving test methods for SARS-CoV-2

Steph H Tan et al. (808–814) make the case for saliva-based tests. 

### Physical activity in childhood and adolescence

John J Reilly et al. (815–824) explain how quantifying inactivity can improve health policies.

